# Breeding novel solutions in the brain: a model of Darwinian neurodynamics

**DOI:** 10.12688/f1000research.9630.2

**Published:** 2017-06-29

**Authors:** András Szilágyi, István Zachar, Anna Fedor, Harold P. de Vladar, Eörs Szathmáry

**Affiliations:** 1 MTA-ELTE Theoretical Biology and Evolutionary Ecology Research Group, Budapest, H-1117, Hungary; 2 Department of Plant Systematics, Ecology and Theoretical Biology, Institute of Biology, Eötvös University, Budapest, H-1117, Hungary; 3 Parmenides Center for the Conceptual Foundations of Science, Munich/Pullach, 82049, Germany; 4 Institute of Advanced Studies, Kőszeg, H-9730, Hungary; 5 Evolutionary Systems Research Group, MTA Ecological Research Centre, Tihany, Hungary

**Keywords:** attractor network, autoassociative neural network, evolutionary search, Darwinian dynamics, neurodynamics, learning, problem solving

## Abstract

**Background**: The fact that surplus connections and neurons are pruned during development is well established. We complement this selectionist picture by a proof-of-principle model of evolutionary search in the brain, that accounts for new variations in theory space. We present a model for Darwinian evolutionary search for candidate solutions in the brain.

**Methods**: We combine known components of the brain – recurrent neural networks (acting as attractors), the action selection loop and implicit working memory – to provide the appropriate Darwinian architecture. We employ a population of attractor networks with palimpsest memory. The action selection loop is employed with winners-share-all dynamics to select for candidate solutions that are transiently stored in implicit working memory.

**Results**: We document two processes: selection of stored solutions and evolutionary search for novel solutions. During the replication of candidate solutions attractor networks occasionally produce recombinant patterns, increasing variation on which selection can act. Combinatorial search acts on multiplying units (activity patterns) with hereditary variation and novel variants appear due to (i) noisy recall of patterns from the attractor networks, (ii) noise during transmission of candidate solutions as messages between networks, and, (iii) spontaneously generated, untrained patterns in spurious attractors.

**Conclusions**: Attractor dynamics of recurrent neural networks can be used to model Darwinian search. The proposed architecture can be used for fast search among stored solutions (by selection) and for evolutionary search when novel candidate solutions are generated in successive iterations. Since all the suggested components are present in advanced nervous systems, we hypothesize that the brain could implement a truly evolutionary combinatorial search system, capable of generating novel variants.

## Introduction

The idea that functional selection on a large set of neurons and their connections takes place in the brain during development ^1– 3^ is now experimentally validated ^4– 7^. As originally portrayed, this process is only one round of variation generation and selection, even if it requires several years. Evolution by natural selection works differently: variants are generated and then selected in iterative rounds. The field of “Neural Darwinism” ^1– 3^ fails to include generation of variants and thus could justifiably be regarded as a misnomer because the process that it describes is not evolutionary in the strict sense ^8^. Evidence indicated that the development of the brain is more “constructivist” ^9^ than pictured by the original selectionist accounts: for example, repeated rounds of neuron addition and loss happen during development ^10^. Structural plasticity (synaptic remodelling) is now known to be a lifelong process with implications for memory and learning ( *e.g.* 11, 12). The addition and deletion of synapses and neurons takes several hours or days ^13^. Our main goal here is to present a proof of principle that bona fide evolutionary dynamics could happen in the brain on a much faster time scale.

Maynard Smith ^14^ identified multiplication, inheritance and variability as necessary features of evolution. In genetic evolution the variability operators are mutation and recombination. If there are hereditary traits that affect the survival and/or the fecundity of the units, then in a population of these units, evolution by natural selection can take place. While this characterization qualitatively outlines the algorithmic aspect of evolution ^15^, concrete realizations require quantitative conditions too: population size cannot be too small (if it is too small, neutral drift dominates over selection ^16^) and replication accuracy cannot be too low (if it is too low, hereditary information is lost ^17^). Note, that this description says nothing about the nature of the units: they could be genes, organisms, linguistic constructions or anything else.

The proper implementation of an evolutionary process within the nervous system could have major implications for neuroscience and cognition ^8, 18– 25^. A main benefit of neuro-evolutionary dynamics would be that it could harness the parallelism inherent in the nervous system and the redistribution of resources at the same time. The latter process means that hopeless variants are thrown away and are replaced in the “breeding space” by more promising ones ^8^. Another important aspect of the process is that it is generative: it could explain where new hypotheses and new policies come from in Bayesian approaches to cognition ^26, 27^ and reinforcement learning ^28– 30^, respectively. Bayesian inference and natural selection are analogous ^31, 32^ in that candidate hypotheses in the brain (the prior distribution) represent a population of evolutionary units, which are evaluated, or selected, based on the evidence. There is a mathematical isomorphism between the discrete-time replicator equation and Bayesian updates ^31^. The likelihood function is analogous to the fitness function and the posterior distribution to the selected population. Relations like this suggest that Bayesian update could be one of the pillars of “universal Darwinism” ^33^. We believe that convincing models for neuro-evolution could empower Bayesian approaches by providing a mechanism to generate candidate hypotheses.

Attractor networks have been used (among others) as models of long-term memory, which are able to complete partial input ^34, 35^. These networks consist of one layer of units that recurrently connect back to the same layer. The recurrent connections can learn (store) a set of patterns with a Hebbian learning rule. Later, if these patterns or their noisy versions are used to provoke the network, it settles on the original patterns after several rounds of activation updates on the recurrent weights (recall), thus stored patterns act as attractors. It is of high importance that the existence of such networks has been experimentally validated in the visual cortex of awake mice by optogenetic methods ^36^. Some versions of the learning rule allow for iterative learning without catastrophic forgetting and enable palimpsest memory. A network with palimpsest memory is able to learn new patterns one-by-one, while sequentially forgetting earlier patterns.

**Figure 1.**
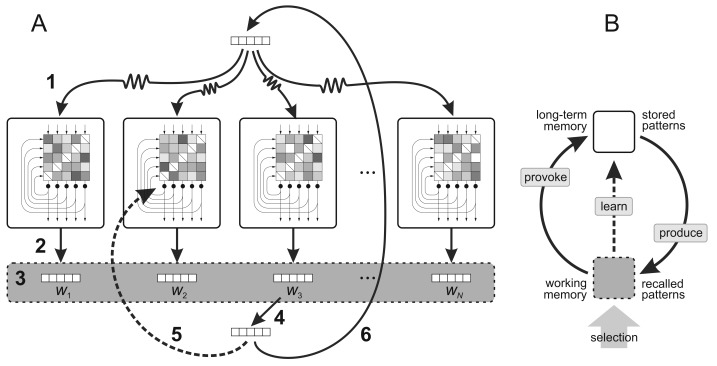
**A) Architecture of multiple attractor networks performing Darwinian search**. Boxed units are attractor networks. Each network consists of *N* neurons ( *N* = 5 in the figure, represented as black dots). Each neuron receives input from the top (1) and generates output at the bottom (2). Each neuron projects recurrent collaterals to all other neurons (but not to itself), forming thus *N* × ( *N* – 1) synapses. The weight matrix of the synapses is represented here as a checkerboard-like matrix, where different shades indicate different connection weights. Selection and replication at the population level is as follows: 1) Each network receives a different noisy copy of the input pattern. 2) According to its internal attractor dynamics, each network returns an output pattern. 3) All output patterns are pooled in the implicit working memory (grey box with dashed outline), where they are evaluated and a fitness *w _i_* is assigned to the *i* ^th^ pattern. 4) The best pattern(s) is selected based on fitness. 5) One of the networks is randomly chosen to learn the pattern that was selected, with additional noise (dashed arrow). 6) The selected pattern is copied back to the networks as input to provoke them to generate the next generation of output patterns. **B) Lifecycle of candidate solution patterns during a cognitive task**. Patterns are stored in the long-term memory as attractors of autoassociative neural networks. When provoked, networks produce output patterns, which are stored in implicit working memory. These patterns are evaluated and selected. Patterns that are good fit to the given cognitive problem can increase their chance to appear in future generations in two possible, non-exclusive ways: 1) selected patterns are used to train some of the networks (learning) and 2) selected patterns are used as inputs for the networks (provoking). The double dynamics of learning and provoking ensures that superior solutions will dominate the system. Erroneous copying of patterns back to the networks for provoking and learning and noisy recall are the sources of variation (like mutations).

In this paper we describe a model that implements evolution of activation patterns in the brain with the help of attractor networks. We see it as a model of problem solving, which is able to generate new candidate solutions to a problem based on past experiences. Any cognitive problem of the brain is encoded by the activity pattern of neurons. We represent neurons as binary units, being able to continuously maintain or suppress firing. A group of neurons therefore has a binary activation pattern. In our model, the units of evolution are these activation patterns, represented as bitstrings. Attractor neural networks can store patterns stably in form of corresponding attractors and are able to recall them given the appropriate trigger ( Figure 1A). This memory allows for heredity, which is indispensable for Darwinian dynamics (in genetic populations memory is the genotype pool). Attractor neural networks can generate new pattern variants in different ways (corresponding to mutation in a genetic system), see below under Discussion. Owing to memory and pattern generation, the possibility of iterated selection over a population of activation patterns becomes feasible. Our approach thus offers a way to implement hereditary dynamics at a faster scale than what could be provided by structural plasticity (cf. 37). This fast-scale dynamics is missing from Edelmanian Neural Darwinism. See the glossary ( Supplementary File 1) and Supplementary Figure S1 for more clarification about the analogy between our model and genetic evolution.

The patterns represent candidate hypotheses or candidate solutions to a problem, which are evaluated based on a fitness function that measures their goodness as a solution. The best patterns (those with the highest fitness) are selected and copied (with variation) back to the networks, which in turn generate the next generation of patterns ( Figure 1B). Stored patterns (attractors) constitute the long-term memory; output patterns constitute the working memory ( Figure 1B). While pattern generation is a simple recall task, which is only able to reproduce previously learnt patterns, the whole system is able to generate new variants due to noisy recall, spurious patterns (see later), noisy copying of patterns, and iterative learning, thus enabling the evolution of novel solutions.

## Methods

**Recurrent attractor networks**. The basic units in our model are attractor networks. Attractor networks are recurrent neural networks consisting of one layer of units that are potentially fully connected. An attractor neural network produces the same (or highly correlated) output whenever the same input is provided (omitting retraining). The pattern that was learned becomes the attractor point of a new basin of attraction, *i.e.* it is the prototype pattern that the attractor network should return. Consequently, an attractor with a non-zero sized basin should also return the same output to different input patterns. However, the amount and type of correlation of input patterns that retrieve the same prototype, *i.e.*, the actual structure of the basin of attraction, is hard to assess, let alone visualize. Still, it is safe to assume that most input patterns correlated with the prototype, produce the same output – the prototype itself.

The Hopfield network is a recurrent artificial neural network with binary neurons at nodes and weighted connectivity between nodes, excluding self-connections. According to the usual convention, the two states of binary neurons are +1 and -1. In our model, a neuron fires (state +1) if the total sum of incoming collaterals is greater than 0. Accordingly, the update rule has the following form:

$$x_{i}(t+1)=\mathrm{sgn}\left(\sum_{j=1(\neq i)}^{N}w_{ij}x_{j}(t)\right).$$

The original Hebbian (covariance) learning rule has the following form (where m is the index of the patterns):

$$w_{ij}^{0}=0,\forall i,j\in \left\{1,2,\ldots ,N\right\},$$

$$w_{ij}^{m}=w_{ij}^{m-1}+\frac{1}{N}\xi_{i}^{m}\xi_{j}^{m}.$$

The Hebb rule is both *local* and *incremental*. A rule is local if the update of a connection depends only on the information available on either side of the connection (including information coming from other neurons via weighted connections). A rule is incremental if the system does not need information from the previously learnt patterns when learning a new one, thus the update rule uses the present values of the weights and the new pattern. The above update rule performs immediate update of the connection weights (“one shot” process; not a limit process requiring multiple update rounds). The covariance rule has a capacity of 0.14 *N* ^38^. However, if the network is trained sequentially, and it reaches its capacity, *catastrophic forgetting* ensues and the network will be unable to retrieve *any* of the previously stored patterns, forgetting all it has learnt.

To overcome this problem and to preserve the favorable properties of the covariance rule (one-shot, local and incremental updating) Storkey has introduced a palimpsest learning scheme ^39^ as follows:

$$\begin{array}{l} w_{ij}^{m}=w_{ij}^{m-1}+\frac{1}{N}\xi_{i}^{m}\xi_{j}^{m}-\frac{1}{N}\xi_{i}^{m}h_{j}^{m}-\frac{1}{N}h_{i}^{m}\xi_{j}^{m}\mathrm{if}i\neq j,\\ w_{ij}^{m}=0\mathrm{if}i=j, \end{array}$$

and

$$h_{i}^{m}=\sum_{k=1}^{N}w_{ik}^{m-1}\xi_{k}^{m}.$$

Using the above rule, the memory becomes palimpsest ( *i.e.* new patterns successively replace earlier ones during sequential learning) with a capacity of *C* = 0.25 *N* (for details and proper definition of palimpsest capacity, see 39). Palimpsest memory is essential for our training regime, where we trained the networks sequentially with one pattern at a time.

An interesting feature of some autoassociative neural networks is the appearance of spurious patterns. In some cases, the network converges to a pattern different from any other patterns learnt previously. These spurious patterns can be the linear combination of an odd number of stored patterns:

$$\xi_{i}^{\mathrm{spur}}=\pm \mathrm{sgn}(\pm \xi_{i}^{m_{1}}\pm \xi_{i}^{m_{2}}\;...\pm \xi_{i}^{m_{s}}),$$

where *S* is the number of the stored patterns ^38^. This effect can be thought of as an effective implementation of a neuronal recombination operator.

In what follows, we will treat a pattern of activations over binary neurons as the unit of selection. In the general case, the adaptive fitness of this pattern may be some complicated function that is contextualized by the current inputs and may or may not be a function of the history of inputs and outputs. To keep things simple, we will just consider the fitness of a pattern in terms of its Hamming distance to some target pattern. This means, we are effectively using selectionist and evolutionary schemes to optimize the connections (and ensuing dynamics) to recover a target pattern.

We present a series of simulations graduating from purely selectionist to evolutionary schemes in the face of a changing environment. In the first set of simulations we preclude variation in transmission over generations to examine the sufficiency of dynamical instabilities in supporting a selective process. This involves taking the outputs of one neural network and using them (after selection) to train another network. In the second set of simulations, we consider evolution proper and the transmission of patterns from generation to generation. Here, the patterns that are transmitted are subject to mutations (and selection) to illustrate the efficiency with which optimal patterns (those with high fitness) emerge.

**Figure 2.**
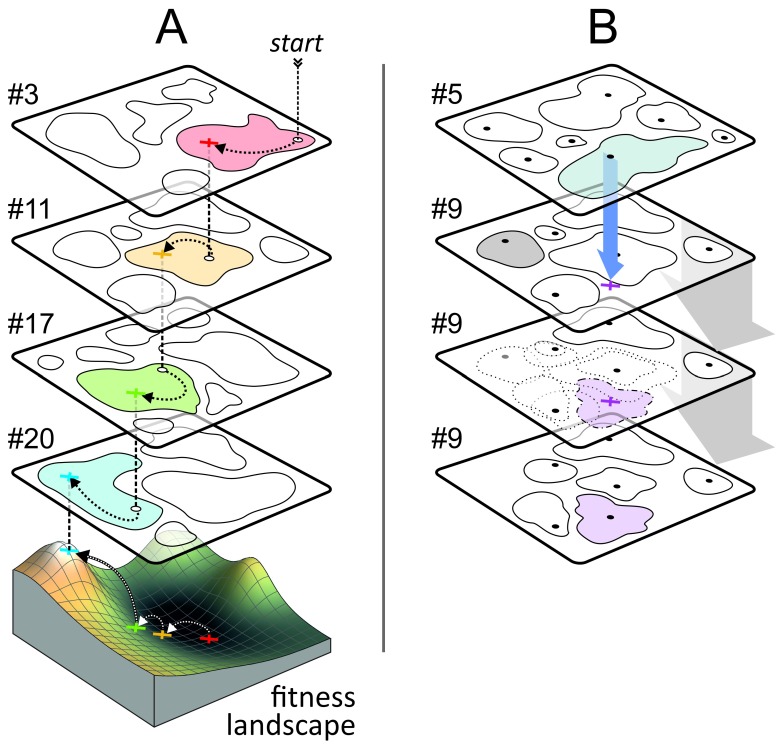
Schematics of attractor networks searching for the global optimum. **A) Four time steps of selection, from top to bottom.** At each step, we only show the network that produces the best output (numbered); the rest of the networks are not depicted. In each time step the networks are provoked by a new pattern that was selected from the previous generation of patterns. Different attractor networks partition the pattern-space differently: blobs inside networks represent basins of attraction. At start, the topmost network (#3) is provoked with an input pattern. It then returns the center of the attractor basin which is triggered by the input. When the output of this network is forwarded as input to the next network (#11), there is a chance that the new attractor basin has a center that is closer to the global optimum. If there is a continuity of overlapping attractor basins through the networks from the initial pattern (top) to the global optimum (bottom), then the system can find the global optimum even without learning. **B) Learning in attractor networks**. Network #5, when provoked, returns an output pattern that is used to train network #9 (blue arrow). As the network learns the new pattern, the palimpsest memory discards an earlier attractor (with the gray basin), a new basin (purple shape) forms around the new prototype (purple ×) and possibly many other basins are modified (basins with dotted outlines). Black dots indicate attractor prototypes ( *i.e.* learnt patterns). With learning, successful patterns could spread in the population of networks. Furthermore, if the transmission of patterns between networks is noisy a network might learn a slightly different version of the pattern, new variation is introduced to the system above and beyond the standing variation. This allows the system to find the global optimum even if it was not used to pre-train any network. The gray arrow in the background indicates the timeline of network #9.

**Selection**. For the selection experiment, we used *N _A_ = 20* structurally identical attractor networks, each consisting of *N* =200 neurons. These networks were initially trained with random patterns plus a special pattern for each with Storkey’s palimpsest learning rule. The 20 special training patterns were as follows. The worst special pattern was the uniform -1, the best special pattern was the uniform +1. Intermediate special patterns had increasing number of +1-s from the left. Fitness was measured as the relative Hamming similarity from the globally best target *O _target_* ( *i.e.* the proportion of +1-s in the pattern). The worst special pattern was presented only to network #1, the second worst to #2, *etc*., while the best special pattern (which was the target pattern) was presented to network #20. In this scenario, no further training occurred ( *i.e*., the dashed arrows on Figure 1 are not there). Assuming that the attractor basins of these patterns overlap among networks ( Figure 2A) the output of one network will be the cue to trigger one or more close special patterns in other networks. The special patterns ensure that there exists a search trajectory leading from the worst to the best pattern, fitness-wise. Starting from any arbitrary initial pattern, if any of the special patterns gets triggered at any time, the system can quickly converge to the optimum – providing the output of each network is delivered to the appropriate network that successively converges on the global optimum.

After initial training, each network received the same random input and generated an output according to its internal attractor dynamics. The output population was evaluated and the best output *O _best_* was selected based on its fitness. Noisy copies (with *μ _I_*, where *μ* is the per-bit mutation probability) of *O _best_* were redistributed for each network as new input for the next generation. These steps were iterated until fitness reached the theoretical optimum ( *i.e*. the system found special pattern #20). The crucial assumption for selection to work is continuity, namely the possibility that the output of one attractor of one network could fall in a different attractor basin of another network returning an output that is closer to the global optimum than the input was (see Figure 1 and Figure 2).

**Figure 3.**
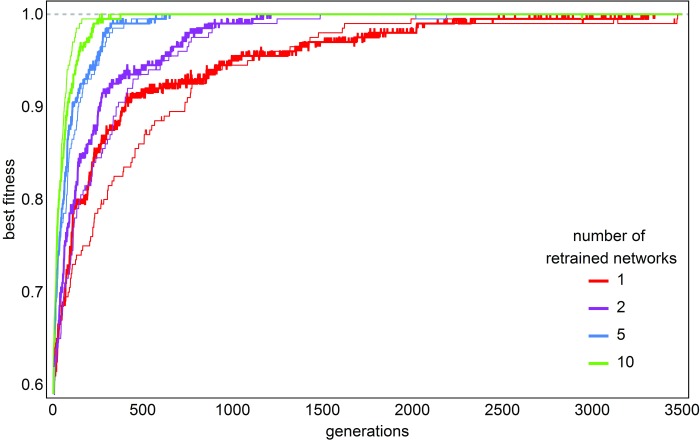
The effect of retraining on the speed of evolution. Lines represent the evolution in four different populations, where a different number of networks were retrained. Each population consisted of 10 networks (see the rest of the parameters under the Methods section). Thin lines: stochastic attractor dynamics; thick lines: simulated attractor dynamics (abstract networks always return the stored attractor prototype that is closest to the actual input, with 0.001 per bit probability noise; capacity to store *C _fix_* = 30 patterns, *μ _O_* = 0.002). Parameters: *N* = 200, *N _A_* = 20, *μ _T_* = 0.01, *μ _I_* = 0.005, elitist selection, keeping the best one only from each output generation; random networks are selected for retraining (never the same in a given generation). Fitness is the relative Hamming similarity to the global optimum.

**Evolutionary optimization on a single-peak landscape**. In contrast to purely selective dynamics, in the evolutionary experiment, networks could learn new patterns during the search process. At the start, each network was trained with a different set of random patterns. The fitness of a pattern is defined as the relative (per bit) Hamming similarity between the given pattern and an arbitrarily set globally best target pattern *O _target_*. The selection process for the actual best output *O _best_* and redistribution of its noisy copies (with *μ _I_* = 0.005) for input was the same as before. Most importantly, the mutated versions (with *μ _T_* = 0.01) of *O _best_* were also used for retraining *N _T_* different networks in each generation (see Figure 1): this forms the basis for the Darwinian evolutionary search over attractor networks, as it allows for replication with variation of (learnt) patterns over networks (thin lines in Figure 3).

We have also analyzed how mutation rates affect the speed of evolution. We measured the number of generations needed for the system to find the globally best target pattern. The previously introduced parameters were used and the mutation rates were varied between 10 ^-4^ and 0.01. We ran each simulation 100 times with different random seeds and measured the average number of generations and standard deviation. We found that only *μ _l_* affects the speed of evolution with an almost constant minimum in a wide range between approx. 0.003-0.007. Mutation rates lower than 0.003 or higher than 0.007 result in rapidly growing number of generations necessary for the system to find the optimum. *μ _T_* does not affect the speed of the evolution considerably (data not shown).

We have compared the search behavior of our system of attractor networks with a simpler model. In this model networks were represented as abstract storage units, which could store exactly *C _fix_* patterns ( *C _fix_* was set to be close to the actual capacity of networks). When such a storage unit receives an input pattern it simply returns the closest (in Hamming distance) of its stored patterns as output, with additional noise ( *μ _O_* = 0.001). The units simulate the almost perfect recall property of attractor networks and effectively approximate attractor behavior. We compared evolution in this simple model with evolution in the system of attractor networks (thick and thin lines in Figure 3).

**Optimization in a changing environment**. In order to test the effect of memory on successive search, we have implemented a periodically changing selective environment, *i.e*., we periodically changed the fitness function. The environment alternated between *E*_1_ and *E*_2_, with a stable period length of *T _E_* = 2000. Each environmental change reset the global optimum: for this scenario, we assumed a uniform +1 sequence for *E*_1_ and its inverse, uniform -1 for *E*_2_ as global optima, and used the relative Hamming similarity as a fitness measure.

**Figure 4.**
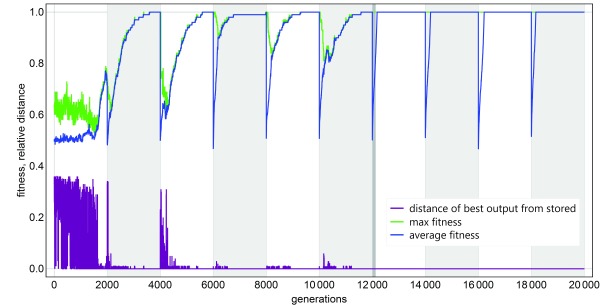
Fitness and recall accuracy over periodically alternating environments. Blue: average fitness; green: best fitness; purple: distance of the best output of the population from the closest one stored in memory (for details, see main text). Grey and white backgrounds represent the changing environment: We alternated two global optimums at every 2000 ^th^ generation. After the 12000 ^th^ generation, we turned off learning (thick vertical line) and set the input to random patterns after each changing of the environment. Parameters: *N _A_* = 100, *N* = 100, *N _T_* = 40, fitness is the relative Hamming similarity to the actual optimum.

In the first phase of the simulation, networks were allowed to learn in each environment for a total of *T _nolearn_* = 12000 generations (three periods per environments). Afterwards, learning was turned off to test the effect of memory. To make sure that the optimal pattern was not simply carried over as an output pattern from the previous environment but was recalled from memory, the input patterns were set to random patterns (instead of inheriting the previous output population) at the start of each new environmental period after *T _nolearn_*. This ensures that the population could only maintain high fitness afterwards in an environment if the optimum was stored *and* could be successfully recalled (see Figure 4). In order to assess the memory of a network, we also measured the distance between the actual best output of the population and the closest one of the set of previously learnt patterns within the same network (as different networks have different training history). A small distance indicates that the network outputs a learnt pattern from memory ( *i.e*. recalls it) instead of a spurious pattern.

For this scenario, we introduced a different selection method (also used in the next section). Each network in the population produces an output according to its internal attractor dynamics and the input it received from the previous generation. From all output sequences one was randomly chosen and mutated ( *µ _R_* = 1/ *N* per bit mutation rate). If the mutant had a higher fitness than the worst of the output pool, the worst pattern was replaced by it ( *elimination of the worst*). Furthermore, the superior mutant was used to train *N _T_* number of different networks. Lastly, the resulting output population is shuffled and fed to the networks as input in the next generation (except when the environment changes and input is reset externally).

**Optimization on a difficult landscape**. To investigate the applicability of this optimization process, we adopted a complex, deceptive landscape with scalable correlation, and also modified the selection algorithm introduced above. We used the general building-block fitness (GBBF) function of Watson and Jansen ^40^. According to the GBBF function, each sequence of length *N* is partitioned into blocks of uniform length *P*, so that *N* = *P B* ( *P*, *B* ∈ **Z** ^+^) where *B* is the number of blocks. For each block, *L* arbitrarily chosen subsequences are designated as local optima, with randomly chosen but higher-than-average subfitness values. The overall fitness *F*( *G*) of a pattern *G* (“genotype”) is as follows:

$$F(G)=\sum_{i=1}^{B}f(g_{i})\;,$$

$$f(g_{i})=\sum_{j=1}^{L}c(g_{i},t_{j})\;,$$

$$c(g,t_{j})=\{\begin{array}{cc} \;w_{j}, & \;\text{if}\;d(g,t_{j})=0\\ {\left(1+d(g,t_{j})\right)}^{-1}, & \;\text{otherwise} \end{array},$$

where *f*( *g _i_*) is the fitness contribution of the ith block in the pattern, *t _j_* is the *j* ^th^ local optimum of length *P* (all *L* different optima are the same for each block in our experiments) with subfitness value *w _j_* > 1, and *d* is the Hamming distance. Consequently, this landscape has many local optima, a single global optimum and a highly structured topology. Furthermore, since there are no nonlocal effects of blocks, each block can be optimized independently, favoring a metapopulation search.

Accordingly, in this experiment, we introduced multiple populations of attractor networks. Each population of *N _A_* attractor neural networks forms a deme and *N _D_* demes are arranged in a 2D square lattice of Moore neighborhood (all of the eight surrounding demes are considered neighbors). Demes might accept output sequences from neighboring demes with a low probability *p _migr_* per selection event; this slow exchange of patterns can provide the necessary extra variability for recombination. These demes correspond to the groups of columns in the brain.

Networks in the deme are the same as those used in previous experiments. However, selection is modeled in a different way, similar to the selective dynamics outlined in 40. In turn, we only give a brief description. Given a deme, each network produces an output according to its internal attractor dynamics and the input it received from the previous generation. Output sequences are pooled and either one or two is randomly chosen for mutation or recombination, respectively ( *i.e. no elitist selection*). With probability *p _rec_*, two-point recombination is performed in the two selected partners, with 1- *p _rec_* probability, a single selected sequence is mutated, with *µ _R_* = 1/ *N* per bit mutation rate. With *p _migr_* probability, the recombinant partner is chosen from another neighboring deme instead of the focal one. Next, the output(s) of recombination or mutation are calculated: if the resulting sequence (any of the two recombinants or the mutant) has a higher fitness than the worst of the output pool, it is replaced by the better one ( *elimination of the worst*). Furthermore, the superior mutant or recombinant was used to train *N _T_* number of different networks within the deme. Lastly, the resulting output population is shuffled and fed to the networks as input in the next generation. Each deme is updated in turn according to the outlined method; a full update of all networks in all demes constitutes a generation ( *i.e.* a single time step).

The GBBF landscape was set up identically to the test case in 40, as follows. For each block uniformly, two target sequences of length *P*, *T*_1_ and *T*_2_, were appointed. *T*_1_ is the uniform plus-one sequence *T*_1_ = {+1} ^*P*^ and *T*_2_ is alternating between -1 and +1 ( *T*_2_ = {-1, +1} ^*P*/2^). According to the fitness rule (Equation 5–Equation 6 in 40 and Equation 1– Equation 3 above), the best subfitness of each block in a sequence can be calculated and the sum of all the subfitness values is the fitness of the global optimum sequence. Thus for sake of simplicity, we used relative fitness values with the global optimum (the uniform +1 sequence) having maximal fitness 1. The sequence(s) with lowest fitness always have a nonzero value.

The source code of all models and data presented in this paper is freely available as a supplement to this paper.

## Results

**Selection**. We should distinguish between two processes: (i) search without learning among the stored patterns to find the best available solution ( *i.e*., selection without step 5 on Figure 1A), and (ii) search with learning: retraining one or more networks with the selected and mutated patterns ( Figure 1A with step 5). The first is a purely selectionist approach because it cannot generate heritable variants, while the second implements Darwinian evolution because learning changes the output behavior of the networks, thus they generate new patterns. First, we analyze the strictly selectionist version, and then the evolutionary version of the model.

In the selectionist version we pre-trained each network with a random set of patterns (excluding the target pattern) and started by provoking them with a different random input. Each network produced an output pattern according to its own attractors and then the best pattern was selected. This pattern was used in turn to provoke the networks in the next generation, and so on. This search has found among all the available stored (pre-trained) patterns the one with the highest fitness; it could not find the global optimum, as the networks were not pre-trained with it and there was no way for new variants to survive in this simulation.

Next, we specifically composed the sets of pre-training patterns: each network was pre-trained with random patterns as before but also with one special pattern. This set of special patterns (in which individual patterns can be ordered according to gradually increasing fitness values) delineate a route to the optimum through overlapping basins of attractors in different networks (see Figure 2A) so that we can test whether in this simplified case the algorithm converges quickly to the optimum. The first population was initiated with the special pattern that was farthest from the optimum. We have found that the selected output gets closer to the optimum in each generation, but the optimization process is saltatory: it skips over many intermediate neighboring special patterns (and thus networks). This is due to the fact that attractor basins of neighboring special patterns were highly overlapping. For example, in Figure 2A, the stored special pattern of network #3 is in the basins of stored special patterns of networks #4–#11, and since the stored pattern of network #11 is closest to the optimum, networks #4–#10 were skipped. A typical sequence of networks generating the actual best output is: #3, #11, #17 and #20 (of 20 networks; for actual parameters, see Figure 2A).

**Evolution**. Learning new patterns as attractors ( Figure 2B) allows networks to adapt to the problem and perform evolutionary search. The results of the evolutionary experiments clearly prove that a population of attractor networks can implement evolutionary search in problem spaces of different complexity ( *i.e*. different levels of correlation and deceptiveness).

***Evolution on a simple fitness landscape***. In this scenario, neither the global optimum nor a route toward it is assumed to pre-exist in the system as in the selectionist experiments: networks are pre-trained only with random patterns. Even under these stringent conditions, we have found that the system can converge to the global optimum, and this convergence is robust against a wide range of mutation rates. Our simplified abstract model, which always returns the stored prototype that is closest to the input, behaves qualitatively the same way (see Figure 3). The speed of convergence to the optimum is mainly affected by the number of retrained networks ( Figure 3): as we increase the number of networks that are retrained we find a faster fitness increase, albeit with diminishing returns. Mutation has an optimal range in terms of the speed of evolution. On one hand, if mutation rate is too low evolution slows down, because there is not enough variation among patterns. On the other hand, if mutation rate is too high it hinders evolution as the offspring is too dissimilar to the parent and cannot exploit the attractor property of the system. When mutation rate is zero, the source of variation is only the probabilistic input-output behavior of the networks due to their asynchronous update and the appearance of spurious patterns when the input is too far from the stored patterns.

While the attractor networks have memory, due to the monotonic, single-peak nature of the fitness landscape there is no need to use it: the system works almost equally well if the networks only store the last training pattern ( *i.e.*, weights are deleted before each learning event). Next, we present experiments where both the attractor property and the palimpsest memory of the networks are used.

***Evolution in a changing environment***. In this experiment we alternated two environments: in every 2000th generation the target pattern (the optimum), against which fitness was measured, was changed. From an evolutionary point of view, this can be perceived as a changing environment, whereas from a cognitive point of view, this procedure simulates changing task demands. Figure 4 shows that the system found and learnt the optima of each of the two environments separately. Then, after generation 12000, we switched off learning. The fact that networks are nevertheless able to recall the target pattern right after the environmental change proves that they use previously stored memories. After we switched off learning, we used random patterns to provoke networks at the first generation of each new environment. A single network that can recall the optimum from the random input is enough to produce a correct output that is amplified by selection for the next generational input, ultimately saturating the population with optimal patterns. This experiment effectively proves that a system of attractor networks can reliably recall earlier stored solution patterns, therefore solving the problem faster in an alternating environment than a system without long-term memory.

**Evolution on a difficult fitness landscape**. The previous evolutionary experiment (where search was on a single-peak fitness landscape with a single population of networks) is a proof of principle of the effectiveness of our evolutionary algorithm. In order to assess the capacity of population search of attractor networks, we introduce a considerably harder fitness landscape with higher dimensionality, where the deceptiveness of the problem can be tuned. The GBBF fitness landscape of 40 provides a method to easily generate scalable and complex landscapes with many deceptive local optima. The complexity of the problem requires the introduction of multiple interacting populations of networks. Though explicit spatial arrangement of the networks is not required to solve the problem, we have nevertheless included it in our implementation to imitate real spatial arrangement of neurons in the brain. Locality allows the exchange of information among neighboring populations ( *i.e*. recombination) that is essential to solve the GBBF problem (or similar deceptive problems) in a reasonable time.

**Figure 5.**
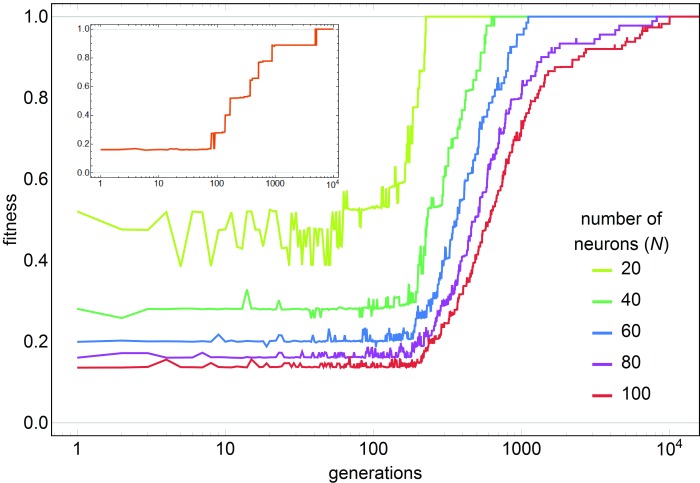
Convergence of the actual best fitness of the metapopulation with increasing problem size *N* on general building block function landscape. Each curve is an average of 10 independent iterations. *N _D_* = 10 × 10 demes, *N _A_* = 10 networks per deme, *N* neurons per network, patterns of length *N* are partitioned to blocks of size *P* = 10 ( *B* = *N*/ *P* blocks per pattern), *p _rec_* = 0.1, *µ _R_* = 1/N, *p*_migr_ = 0.004, *N _T_* = 5. Note the logarithmic *x* axis. Inset: single simulation at *N* = 80, *B* = 8 (other parameters are the same). Plateaus roughly correspond to more and more blocks being optimized by finding the best subsequence on the building-block fitness landscape.

We have investigated the performance of search in a metapopulation with different problem sizes (pattern lengths; see Figure 5). Results indicate that despite the vastness of the search space, the metapopulation is always able to converge to the global optimum, given enough time. The most complex landscape of 100-bit patterns is of size 2 ^100^ with one global optimum and a huge number of local optima. The metapopulation consists of 10 ^5^ neurons (100 populations of 10 networks each with 100 neurons per network) and can find the single global optimum in ~10 ^4^ time steps. The limit of further increasing the problem size is in the computational capacity of our resources.

## Discussion

**Summary of results**. Attractor networks can be used to implement selection, replication and evolutionary search. As a proof of principle, we showed that attractor networks find the global optimum in a purely selectionist model ( *i.e.* without learning) if they are pre-trained with attractors that overlap in their basins and lead to the optimum. The population can effectively select over the standing variation of all stored patterns and find the trajectory to the (single) peak of the fitness landscape (see Figure 2B). Furthermore, if learning is allowed during search, the relative frequency of good patterns (those closer to the optimum) can be increased by re-training networks with such patterns, so that they are stored in the long-term memory in more copies ( Figure 3). Overwriting an older memory trace with a new pattern corresponds to a copying operation, potentially with mutations. A particularly interesting aspect of a population of attractor networks under the given coupling is that even if learning is not erroneous, the Lamarckian nature of inheritance of patterns (as output → memory → output; see Figure 1B) means that there is room for heritable variation to emerge at other stages of the cycle, thus implementing Darwinian dynamics.

The explicit benefit of memory manifests in a periodically changing environment. In a single, stable environment, memory is not very useful because the attractor property acts against exploring variation, and networks might be even slower than gradient hill-climbers ( *i.e.* searchers who generate mutant output blindly and only take a step on the landscape if the output is better than the input). However, in a periodically changing environment, attractor networks with memory are able to recall the actual global optimum if ** they have already encountered the given environment in the past and stored its optimum; hill-climbers or naïve attractor networks lacking the necessary memory would have to perform the search all over again. Attractor network can recall the appropriate pattern even if there is no initial cue for the population to know which environment it is in. A network with memory can complete a partial cue and thus can recall the global optimum. After learning has ceased, it is enough to only have a few networks in the population that can recall the optimum from random cues that are accidentally close to the actual optimum (maximal fitness is immediately 1 in the population, see green curve in Figure 4). Selection then simply amplifies these optimal output patterns in the population (as output is fed back as input in the next generation) until all networks receive optimal patterns as input. At that point, average fitness also reaches the maximum ( Figure 4, blue curve). A network without memory would have to search for the new optima in each environment over and over again, finding the whole uphill path on the landscape.

We also proved that a metapopulation of attractor networks can successfully optimize on a complex, correlated landscape of higher dimensionality. This is a notoriously hard optimization problem (cf. 40) as hill-climbers can easily get stuck in deceptive local optima. The spatial organization of attractor networks resembles the spatial organization of neural structures of the cortex and it allows parallel optimization of subproblems. By this independent optimization of solution parts, local populations can exchange successful partial solutions with each other and form recombinants that are necessary to solve such complex problems in reasonable time.

**Evolutionary context**. We have chosen attractor networks to demonstrate Darwinian neurodynamics because (i) the search for existing solutions uses the same architecture for generating, testing and storing novel ones; (ii) stored patterns help evolutionary search by readily employing related past (learnt) experience, and (iii) the particular choice of Storkey’s model naturally results in some new recombinant patterns. This is an important point because, as we know from population genetics ^41^, recombination speeds up the response to selection by creating new variants ^42^. For a non-exhaustive mapping between population genetics ad Darwinian neurodynamics (see Supplementary File 1 and Supplementary Figure S1).

Our choice of implementation of attractor networks, following Storkey’s model ^39^, is based on three important aspects: (i) it has palimpsest memory, so that it can learn new and forget old patterns without catastrophic forgetting, as happens in Hopfield and other networks; (ii) its attractors are roughly of equal size and are well-structured according to a Hamming distance measure, and (iii) unlike most other neural networks, it is able to store correlated patterns. The downside is that these networks require full connectivity, which is neuronally unrealistic. However, its functional properties reflect well what we know of long-term memory in the brain, which is enough for a proof or principle of an evolutionary implementation of neuronal function. To our knowledge no model exists in the literature that could satisfy all requirements above and that, at the same time, work with diluted connectivity ^43^.

It is important to clarify that the units of evolution in our proposed Darwinian neurodynamics system are the activation patterns: they are copied potentially with errors, selected and amplified. However, patterns live in two stages: in the working memory and in the long-term memory ( Figure 1). This implies different inheritance methods (routes to pass on information) from what is expected in a purely genetic system. Changed attractor prototypes imply changed outputs, just like a mutated genotype implies a different phenotype. However, in our proposed system, changes made to output patterns (by mutation) can also be “inherited” by the stored attractor prototypes via learning. Furthermore, there is another difference in the dynamics, explained in turn.

Darwinian evolution is often described as a parallel, distributed search on a fitness landscape ^8^. The population, as an amoeba, climbs the peaks of the landscape via iterative replication and selection in successive generations. The attractors, however, impose a different mode of evolution, because they simply return the prototype pattern closest to the input, even if it is less fit than the input pattern itself. Consequently, attractor networks work against variability and slow down hill-climbing. However, attractor networks resist fitness increase only half of the time on average; the other half of the time they effectively push inferior patterns uphill in the fitness landscape at a speed much higher than that expected for the same (reduced) amount of genetic variation. Consequently, attractor networks can facilitate evolution ^21^.

We stress the importance of evolutionary combinatorial search. In cases where *ab initio* calculation of molecular functionality is impossible, artificial selection on genetic variants has proven to be an extremely useful method to generate molecular solution to functional problems, as experiments on the generation of catalytic RNA molecules (ribozymes) illustrate (see 44 for a recent review). By the same token when a brute force numerical calculation of a combinatorial functionality problem is impossible for the brain, given the adequate architecture it could (and we suggest it does) use an evolutionary search mechanism, as shown in this paper.

**Implementation in the brain**. It is of primary importance that all the components in our ‘algorithmic diagram’ ( Figure 1) can be implemented by mechanisms known in the brain. It is likely that the cortical hypercolumn ^45^ behaves like an (at least one) attractor network. The reciprocal connections between the long-term and working memory networks are assumed to be like those in Figure 3 in 46. We propose that, first, the reinforcing signal from the basal ganglia via the thalamus keeps active the good candidate pattern solutions in the rewarded auto-associative network and, second, that the latter sends a copy of the active pattern to (unconscious) working memory for eventual redistribution. When there is an increase in the quality of a solution (fitness increase) or when a local or global optimum is reached, the central executive elicits the transmission of a copy of the solution to the conscious memory ^47^.

Our proposed mechanism relies on information transmission of patterns between cortical groups and relevant subcortical structures with variation, and in this way it differs from all previous models. A discussion of timescales is in order. Without learning newly generated variants, the selective mode would require about the same time as suggested by 48 as the “cognitive cycle” (based on data), but without perception at the beginning of the cycle, *i.e.* it would be in the 160–310 ms range. In the evolutionary mode, learning of new variants is required which would take more time. A conservative estimate for the reliable expression of changed synaptic weights is between seconds to minutes ^49, 50^. The second scale would allow several dozen cycles/generations per minute—a very good number in artificial selection experiments. A more accurate estimate of timing will require a fully neuronal model of our proposed mechanism.

Copying of information is necessary for evolutionary processes: this is where previous approaches ^22– 24, 51^ have been too artificial. There are four well known instances where scholars invoke copying of information in the brain: (i) the transfer of information from short to long-term memory ^35, 52, 53^, (ii) the transfer of information from specialized cortical areas to working memory ^54^ or to the global workspace ^55, 56^ and, finally (iii) the possible copying of patterns from working memory to conscious processing ^57^. Undoubtedly, all these approaches require accurate information transfer between different brain components ^58^.

In the context of Darwinian neurodynamics, inheritance is replaced by learning. De Vladar and Szathmáry 21 showed that the rate of “mutation” in spiking diminishes as there is convergence to the optimum. In other words, as the population of attractors approaches the solution, the rates of mutation decrease, since the need to generate new hypotheses decreases. This type of adaptive mutation rate results in what is known as “evolvability” ^59^ and in Darwinian neurodynamics it comes for free: while selection ensures that more accurate hypotheses are implemented, learning through the strengthening of connections between simultaneously active neurons decreases the rate of variability. Hence, the crucial effect in neurodynamics comes from the built-in mechanism that effectively implements second-order selection by adapting the mutation rate according to the quality of the standing hypotheses.

There are three sources of variation in our system: (i) due to the finite size of our networks and asynchronous update of the neurons, the output patterns show some variation even if a network is repeatedly provoked by the same input pattern (noisy recall), (ii) acknowledging the noisiness of neuronal transmission we introduce variation akin to mutations when patterns are transmitted among the blocks of the model (we mutate the patterns with mutation rate μ), and finally (iii) we have realized that “spurious patterns” ^38^ emerge as by-products of previous training patterns and they might act as (new) recombinants of learnt patterns that facilitate the evolutionary search.

The non-conscious or implicit working memory, which has received considerable attention lately ^47, 57^ is crucial for our proposed mechanism. Irrespective of whether working memory overlaps with the conscious domain ^60^ or not ^57^ (in the latter case a ‘conscious copy’ must be sent from working memory to conscious access), the important factor is that the bound on the number of patterns that can be held in the *unconscious* part of the working memory is larger than that of the conscious working memory ^60^. In other words, our mechanism suggests that the total storage capacity of the unconscious network population is much higher than that of the conscious one. Crucially, there is support for this requirement: there is evidence that the central executive function of working memory is not restricted to the conscious domain either ^47^. The relatively large capacity of (unconscious) working memory can hold not one, but several patterns selected by the cortex-striatum-basal ganglia loop. This type of selection can be realized by a winner-share-all (WSA) mechanism ^61^.

The latter point requires special attention. The reader is referred to the recent review by 62 on models of action selection and reinforcement learning. We wish to make a few critical points in this regard. First, as we are considering problem solving that unfolds its capacity online, there is no reason to select one pattern, since the interim patterns are not alternative actions but only candidate solutions. They can be turned into actions sometimes during, or only at the very end, of the evolutionary search. Weak lateral inhibition within the evaluation mechanism enhances value differences in selection, but a single winner is not selected ^61, 62^. Second, parallelism of the evolutionary approach loses considerable power if the evaluations are not done in parallel, and if poor solutions cannot be replaced by better solutions in the storage. (In a subsequent study we shall show that the number of parallel evaluations are allowed to be considerably smaller than population size but also that purely serial evaluation of candidates is a killer). Third, it is perfectly possible that the WSA part is implemented by the cortex rather than the striatum (cf. 62): we are agnostic on this point for the time being. (Admittedly that option would require a different version of the full model). Fourth, we maintain that parallel survival of a number of candidates should happen, and the mechanism for this might have evolved with selection for complex (offline) problem solving. Fifth, it is well possible that WSA is gradually reduced towards WTA (one winner takes all) during evolutionary search in the brain: this would also guard against premature convergence early and fast convergence towards the end of the search.

To sum up, we have seen that a process analogous to natural selection can be rendered into a neuronal model employing known neurophysiological mechanisms. Now we discuss relations to some other publications and outline future work.

**Related work**. Several examples show that evolution with neurodynamics can be more powerful than either of the components alone. Fernando *et al*. ^25^ proved that the path evolution algorithm – which includes both elements of structural plasticity ^63, 64^ and iterative generation of variation – is more powerful in several regards than classical genetic algorithms. Fernando *et al.* have also shown that replication combined with Hebbian learning is more powerful than classical natural selection in a model of mechanistic copying of binary activity ^22^. De Vladar and Szathmáry provided proof that the synergy between selection and learning results in increased evolvability; also they pointed out that synaptic plasticity helps escaping impasses and build circuits that are tailored to the problem ^21^. Finally, in a recent model Fernando and his colleagues have used autoencoders for the generation of “neuronal genotypes” ^65^. Since autoencoders produce compressed representations of the input, we expect them to successfully replace the identity function ( *i.e.* bit by bit copying, as in DNA replication). Indeed, applying this neural component within the context of a genetic algorithm turned out to be rewarding.

Unless the envisaged information transfer is accurate enough in space and time, the evolutionary dynamics breaks down. Similar to genetic evolution, where the error threshold ^17^ had to be raised before long informative genomes could arise by evolving adaptations for more accurate copying ^66^, in the neuronal context the element of accuracy was raised by Adams ^18^. In his “Hebb and Darwin” paper Adams talks about synaptic replication and synaptic mutation as important ingredients for a Darwinian view of the brain. Synaptic replication means either the strengthening of an existing synapse, or the making of a new synapse between two neurons that already have one synapse between them. Adams’ is an important insight: evolutionary dynamics does not need copying for selection (scoring or strengthening is enough), but it needs copying with errors to test the new variants against the others. Synaptic mutation happens when a neuron grows a synapse towards a neighboring neuron with which previously it had no contact. Interestingly, these thoughts preceded the burst of interest in structural synaptic plasticity (SSP, ^63, 64^). Following his expansion-renormalization model for SSP ^67^ Kilgard observes that when SSP is used for learning something new, this could be regarded as a Darwinian mechanism ^37^, as it generates and tests variations in successive rounds, based on what is already there (unlike the original models of “neural Darwinism”). Kilgard’s mechanism has not been formalized yet (although see 21), but the path evolution model ^25^ bears some relationship to it.

We share the view of Eliasmith ^54^ that the cortex/basal ganglia/thalamus/cortex loop plays a crucial role not only in elementary action selection but also in symbolic reasoning. We conjecture that non-primate animals (in particular mammals and birds) employ the same (or at least an analogous) loop in order to retrieve old and to innovate new solutions, in a similar way as we have shown using our elementary model.

Another view to which we feel strongly related to is that of Bayesian models that advocate “theory learning as stochastic search in a language of thought” ^27^. We are reasonably confident that we have found a candidate mechanism for the search process. If true, the rugged learning landscape in Figure 3 of 27 can be directly interpreted as the fitness landscape of our neuro-evolutionary model. A task for the future is to work out the explicit relations in detail. We note again the formal link between Bayesian inference and evolutionary selection ^31, 32^ mentioned in the Introduction. This has further implications for the Bayesian brain, which indicate that our neuro-evolutionary framework is not only a metaphor, but works in a way that is at least equivalent to an evolutionary search. From the Bayesian perspective, the brain weights a series of candidate hypotheses according to their likelihood. From the evolutionary perspective, this update is interpreted precisely as the action of selection on the standing round of hypotheses, with the next round or generation given by the posterior probability of these hypotheses. Moreover, the selective likelihood has been shown in previous models of population genetics to be analogous to a free energy function that is minimized during evolution ^68– 71^. These models incorporate mechanisms for generating variability, a component that seems to be absent in previous theories of Bayesian brains. But as evolutionary models show, it is utterly essential to account for mechanisms that create new variation, or, in the neurodynamical context, new hypotheses.

Our mechanism ( Figure 1) could in principle implement, with appropriate modifications, an estimation of distribution algorithm (EDA). The population-based incremental learning (PBIL) algorithm consists of the following steps ^72^: (i) Generate a population from the probability vector; (ii) Evaluate and rank the fitness of each member; (iii) Update the probability vector based on the elite individual; (iv) Mutate the probability vector; (v) Repeat steps (i–iv) until a finish criterion is met. EDA can work better than copying algorithms making it an interesting line to pursue.

Future work will be to link our recurrent model with the feedforward autoencoder model of Fernando *et al*. ^65^, since the latter can generate interesting genotypes (better substrates for selection) due to the emerging compressed representations of the inputs.

As two experts aptly remark: “The Bayesian brain falls short in explaining how the brain creates new knowledge” ( 73 p. 9). We suggest that neuronal evolutionary dynamics might serve as a remedy.

## Data and software availability

Zenodo: István Zachar at doi: 10.5281/zenodo.154113 ^74^; András Szilágyi at doi: 10.5281/zenodo.803989 ^75^.

The algorithm described in the paper is also available on GitHub at https://github.com/IstvanZachar/Neurodynamics and at https://github.com/andszilagyi/darwinianneurodyn.

## Supplementary material

**Supplementary File 1. Glossary of terms, summarizing the important evolutionary and neurobiological concepts and explaining the analogy between genetic and neuronal Darwinism.**

**Supplementary Figure S1. Analogy between genetic evolution and the attractor network-based model of Darwinian Neurodynamics.** **A**) genetic evolution; **B**) attractor network-based model of Darwinian Neurodynamics. The encoded information (genotypes or attractor networks) express a trait (a phenotypic component in case of genetics; an output pattern in case of the model). Selection acts over these expressed traits, and the new generation’s distribution of encoded information depends on this selection process. In genetic evolution, the genotype is inherited at generation t+1 with some mutations. In our neurodynamical model, some attractor networks (and attractors) are left unaffected, and some are altered by training them with one of the selected and mutated patterns. For more information about the analogy of Darwinian inheritance systems, see 76.
