# Supplementary material for: Breeding novel solutions in the brain: a model of Darwinian neurodynamics
Source: F1000Res. 2017 Jun 29;5:2416. Originally published 2016 Sep 28. [Version 2] doi: 10.12688/f1000research.9630.2 (PMC5130073; doi:10.12688/f1000research.9630.2)
Supplement: Supplementary file 1 [file f1000research-5-12941-s0000.tgz › 9f1cc18e-bf35-4f82-a736-38b9a2c3dac2.pdf]

Glossary

Attractor network: Neural network model of associative memory that can store, complete and retrieve patterns that it previously learned. Attractor networks encode states into attractors, meaning that for many input patterns, the subsequent iteration of the network will produce the same set of outputs.
Breeding: Producing new output patterns by provoking the attractor networks with input patterns.
Catastrophic forgetting: Major or total loss of memory content of a neuronal network due to overload of its capacity.
Evolution: Heritable changes of biological traits in a population through successive generations. In the context of our model, evolution means that the composition of output patterns changes from one generation to the next.
Evolutionary dynamics: Response of the composition of a population specified by the forces and factors that provoke changes in heritable characters.
Fitness: Potential of an output pattern to be transmitted to the next evaluation round; in the context of this paper it is measured as the Hamming distance from the target pattern (the solution).
Generation: The collective of individuals that exist at a particular step in the line of descent of a population. In the case of neurodynamics, the line of descent should be understood as evaluation rounds, i.e. every time a population of outputs is produced.
Heritable: A trait is heritable if it can be passed down genetically from parent to offspring.
Lamarckian inheritance: The idea that acquired traits can be passed down from parent to offspring.
Memory: When a neural network stores a pattern in its memory, it means that it is able to produce the pattern as an output pattern, when it is provoked with a noisy version of the pattern.
Metapopulation: An ensemble of populations, interacting at some level, where each of them has its own dynamics according to local environment.
Mutation: In the context of our model, mutations are random changes in the bit-string patterns due to intrinsic input-output erroneous behaviour of the networks and/or incorrect copying of the patterns across networks and iterations.
Neutral drift: Random changes in a population due to finite size sampling.
Palimpsest memory: When a neural network has palimpsest memory it means that it can go through subsequent training rounds without catastrophic forgetting; instead, it forgets previously learnt patterns gradually as it learns new ones, maintaining its memory capacity at a certain level.
Patterns: Sequence of bits (binary strings) that encode a hypothesis or potential solution.
Recombination: A variation-generating process by means of combining segments of two or more bit-strings.
Replication: Process whereby copy of a biological type or structure (e.g. a genotype, a bit-string, etc.) is generated.
Selection: In the context of this model, selection is the process by which the patterns are chosen to be used as input patterns in the next generation either for re-training the attractor networks or to provoke them.
Structural plasticity: The ability of a neuronal system to modify its synaptic connectivity during the process of learning.
Training: A process where the training rule is used to modify the weights of a neural network based on the training pattern(s). Pre-training happens before the first generation of output patterns is produced; all subsequent training is called retraining in this paper.
Units of evolution: A replicative structure that is subject to natural selection and heritable variation.
Variation (heritable): Changes in a character that are transmissible from parent to offspring.
