# Supplementary material for: Breeding novel solutions in the brain: a model of Darwinian neurodynamics
Source: F1000Res. 2017 Jun 29;5:2416. Originally published 2016 Sep 28. [Version 2] doi: 10.12688/f1000research.9630.2 (PMC5130073; doi:10.12688/f1000research.9630.2)
Supplement: Supplementary file 2 [file f1000research-5-12941-s0001.tgz › 8290cb77-0152-4adc-90d6-fc6a8e291115.pdf]

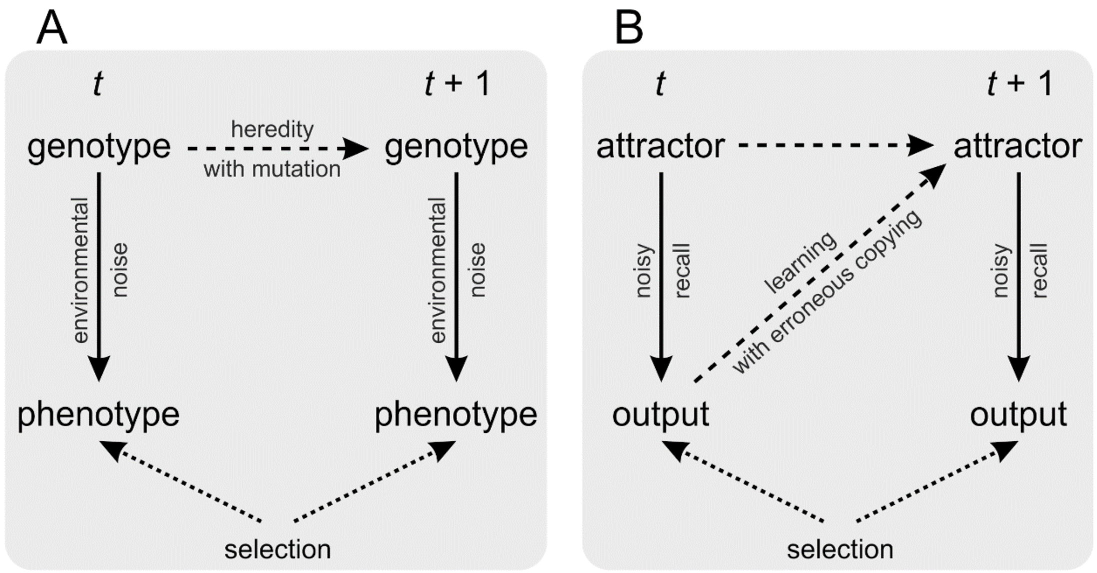

Supplementary Figure S1. Analogy between genetic evolution and the attractor network-based model of Darwinian Neurodynamics

A) genetic evolution; B) attractor network-based model of Darwinian Neurodynamics. The encoded information (genotypes or attractor networks) express a trait (a phenotypic component in case of genetics; an output pattern in case of the model). Selection acts over these expressed traits, and the new generation's distribution of encoded information depends on this selection process. In genetic evolution, the genotype is inherited at generation t+1 with some mutations. In our neurodynamical model, some attractor networks (and attractors) are left unaffected, and some are altered by training them with one of the selected and mutated patterns. For more information about the analogy of Darwinian inheritance systems, see Zachar 2011.
