# Supplementary material for: Breeding novel solutions in the brain: a model of Darwinian neurodynamics
Source: F1000Res. 2017 Jun 29;5:2416. Originally published 2016 Sep 28. [Version 2] doi: 10.12688/f1000research.9630.2 (PMC5130073; doi:10.12688/f1000research.9630.2)
Supplement: Peer review history

# Peer review: Referee response for version 2

**DOI:** 10.5256/f1000research.12941.r23918

**Published:** 2017-07-17

**Authors:** László Acsády

**Affiliations:** 1 Laboratory of Thalamus Research, Institute of Experimental Medicine, Hungarian Academy of Sciences, Budapest, Hungary

I have read this submission. I believe that I have an appropriate level of expertise to confirm that it is of an acceptable scientific standard.

# Peer review: Referee response for version 2

**DOI:** 10.5256/f1000research.12941.r23919

**Published:** 2017-07-03

**Authors:** Stuart J. Edelstein

**Affiliations:** 1 Ecole Normale Supérieur, Paris, France

A timely article now fully acceptable.

I have read this submission. I believe that I have an appropriate level of expertise to confirm that it is of an acceptable scientific standard.

# Peer review: Referee response for version 2

**DOI:** 10.5256/f1000research.12941.r23917

**Published:** 2017-06-30

**Authors:** Karl Friston

**Affiliations:** 1 Wellcome Trust Centre for Neuroimaging, University College London, London, UK

Many thanks for responding to my previous comments and congratulations on a very interesting piece.

I have read this submission. I believe that I have an appropriate level of expertise to confirm that it is of an acceptable scientific standard.

# Peer review: Referee response for version 1

**DOI:** 10.5256/f1000research.10377.r17462

**Published:** 2016-11-25

**Authors:** László Acsády

**Affiliations:** 1 Laboratory of Thalamus Research, Institute of Experimental Medicine, Hungarian Academy of Sciences, Budapest, Hungary

This is a truly enlightening and thought provoking paper which utilizes Darwinian logic to explain neuronal network dynamics. The manuscript is a nice example of how approaches in a different discipline (population genetics) may yield fresh insights into age old problems of neuroscience.

 I have the following notes, suggestions and questions to the authors.

 Introduction:

1. One drawback of the paper is that the parallelism between genetic and neuronal Darwinism is not made clear right at the onset. We should be aware of what the authors mean by parent, offspring, multiplication, mutation, selection in case of neuronal activity right from the beginning in order to follow the logic of the paper.

Results:

1. I miss the clear demonstration of the “Selection” experiments showing that it is not able to find the global optimum (e.g as an additional panel to Fig 2). I also think we need some form of quantification here, how many simulations were run, how significant the result was…etc.etc
2. I also miss the formal demonstration of the effect of mutation rate on the speed of evolution. Since this is a crucial concept, I would dedicate a separate figure for that.
3. I would like to see, how implementing palimpsest memory affects the performance of the model and how this depends on whether the system use dense or sparse coding. Presently this is only briefly mentioned in the Method section, but since this may have important implications it may be good provide some more details. Intuitively, more sparse coding may tolerate the lack of palimpsest memory.
4. Neuronal noise considered as “mutation” in the model is enlightening. Still, I feel there are some basic differences here. During evolution genetic mutations can get stabilized when they reach the global optimum whereas neuronal noise is inherent to the system and not necessary change with evolution. Can it be demonstrated or is there any evidence that neuronal noise decreases as the system approaches the optimum state?
5. What were the connection weights of the recurrent (i.e. new input) patterns relative to the weights of the local, autoassociative connections? Can the model perform better/worse by changing the relative weights of these connections? Note that many original autoassociative models worked with a “detonator” synapse as an input and weaker local connections ^1^.

Discussion:

1. I would not necessarily constrain the model to implicit memories. I think “implicit” here refers to the unconscious effort to recall the best target pattern not to type of memory item to be recalled. The term “implicit memory” evokes mainly procedural memories and indeed the authors place the model in the cortex-basal ganglia loop. I don’t see why recall of an episodic memory trace by the CA3 recurrent network cannot follow the same evolutionary logic even though hippocampal memories are not considered as “implicit”.
2. In the cortex-basal ganglia-thalamus loop, it is not really known how exactly the cortical output will affect the return signal from the thalamus but, in any case, the signal goes through significant dimension reduction ^2^ and the final output of basal ganglia may also affect thalamic firing in different ways ^3^ (i.e it is “mutated” a lot). The question is, how the properties of the model network changes if the final output is not directly fed back to the system but undergoes various (but consistent) signal transformation.

Minors:

1. I miss the definition of “best pattern”. Can this term be equated with “pattern with highest fitness”?
2. I would also support a short glossary with the neurobiological relevance of the crucial ecological concepts (fitness, generation, landscape, mutation).
3. I guess subheadings in the Results section are not appropriate. “Selection” and “Evolution” are the two main subheadings and all the others are subsections of the “Evolution” section.

I have read this submission. I believe that I have an appropriate level of expertise to confirm that it is of an acceptable scientific standard, however I have significant reservations, as outlined above.

## References

[1] TrevesARollsET: Computational constraints suggest the need for two distinct input systems to the hippocampal CA3 network. *Hippocampus*.1992;2(2) : 10.1002/hipo.450020209 189-99 10.1002/hipo.450020209 1308182

[2] Bar-GadIBergmanH: Stepping out of the box: information processing in the neural networks of the basal ganglia. *Curr Opin Neurobiol*.2001;11(6) :689-95 1174101910.1016/s0959-4388(01)00270-7

[3] GoldbergJHFarriesMAFeeMS: Basal ganglia output to the thalamus: still a paradox. *Trends Neurosci*.2013;36(12) : 10.1016/j.tins.2013.09.001 695-705 10.1016/j.tins.2013.09.001 24188636PMC3855885

# Response

**Published:** 2017-06-17

**Authors:** István Zachar

**Affiliations:** ELTE, Hungary

We are grateful for the review of László Acsády, and his suggestions to improve our paper. We refer to the points raised by our Reviewer by a hashmark (#). Please also see our answers to the other Reviewers for some relevant answers.

 Introduction #1. There is limited similarity only between neural Darwinism and the model and thoughts presented in this paper. In both cases “neuronal groups” are important in terms function, although the term “neuronal assembly” is more traditional and functionally relevant. Synapses are important inasmuch as they contribute to the functionality of the assembly. There is a form of reentry in both models but they play very different roles: in our case it means that the same or variant information is fed back, after evaluation, to the same population of assemblies and get stored in multiple copies. It is this multiplication component that supports “neuronal replication” of candidate solutions.

 A note is in order about the search mechanism in the space of candidate solutions. In the classic Deheane-Changeux model for prefrontal cortex functionality in terms of action production and selection on the example of the Wisconsin card sorting test [1], search for new candidate solutions is random and their evaluation is strictly serial. A non-random but still serial search process, for which there is neural evidence, is chaotic itinerancy [2-4]. Except for the learning phase our attractors are locally stable, classical ones. In contrast, chaotic itinerancy rests on so-called “attractor ruins” from which there is always the possibility of dynamical escape in certain directions via transient chaotic bridges. Since these attractor ruins are deterministic entities, escape directions are not random. The pertinent question is whether these directions are random relative to those leading to better candidate solutions. One can imagine that synaptic plasticity combined with reinforcement learning might strengthen certain escape routes with experience.

 An explanatory table and an accompanying figure were added to the text to guide the reader among the terms we use and to explain the relationship we imply between genetic and neuronal Darwinism.

 Results #1. In case of Selection experiments the system can always find the global optimum, the best special pattern (it was trained to network #20). The overlapping basins of the series of special patterns among networks guarantee that there is a trajectory from the basin of any one of the special patterns to the best special pattern. All simulations were started from noisy copies of a random input pattern similar (in Hamming-distance) to the worst special pattern. We ran 1000 independent simulations, the average number and variance of the rounds needed to converge to the optimum were 3.5± 0.64.

 Results #2. A new figure and a few sentences were added to demonstrate the effect of mutation rate on the speed of evolution.

 Results #3. Palimpsest memory is essential to our model. Because of the repeated retraining of the networks with the selected patterns, without palimpsest memory all networks – even if the patterns are sparse – sooner or later would reach catastrophic forgetting. Consequently, palimpsest memory is necessary for evolutionary processes. We used dense coding for the reason of computational time.

 Results #4. In the present state of the model, the two types of noise (m _l_ and m _T_) are considered to be constant. Using decreasing m _l_ with increasing fitness does not change the qualitative outcome of the evolutionary process (results not shown). Changes in m _T_ do not affect the convergence too much, see the answer for question #2 above.)

 Results #5. To be in line with Storkey’s original model we used similar weights to the recurrent patterns and autoassociative connections. In the present state of the model, we have not tested the palimpsest behavior and the retrieval ability with other than 1:1 relative weights.

 Discussion #1, #2. We have presented our model as potentially realisable by the cortex-basal ganglia-thalamus cortex loop. Actually, noting the massively parallel processing in the brain in general, added to the evaluative function of the mentioned loop, we would be surprised if no true evolutionary behaviour could be found during complicated problem-solving tasks. But we agree that it is not necessary to restrict the proposed cognitive architecture to this concrete loop: recall of an episodic memory trace by the CA3 recurrent network [5] can in principle follow the same evolutionary logic, even though hippocampal memories are not considered as “implicit”.

 A deeper question considers how “informational reentry” in the Darwinian sense can be realized by the cortex-basal ganglia-thalamus-cortex loop. In this loop it is not really known how exactly the cortical output will affect the return signal from the thalamus but, in any case, the signal goes through significant dimension reduction [6] and the final output of basal ganglia may also affect thalamic firing in different ways ([7], i.e. it is “mutated” a lot). The question is, how the properties of the model network change if the final output is not directly fed back to the system but undergoes various (but consistent) signal transformation. We think that the key to our kind of mechanism to work is exactly the consistency of signal transformation. A variant mechanism might look like this: the thalamus pointedly activates those cortical networks where the best (few) activity pattern(s) came from (as before, the number of candidate patterns “kept alive” depends on the stringency of selection in the striatum). These could in turn spread in the cortex through the horizontal connections among local attractors. Passing of information from attractor network to another is a common element in many relevant dynamical models [8,9].

 Minor points were all corrected according to Reviewer’s suggestions.

 References:

 1 Dehaene S, Changeux JP. The Wisconsin Card Sorting Test: Theoretical Analysis and Modeling in a Neuronal Network. *Cerebral Cortex*. 1991;1(1):62. http://­dx.doi.org/­10.1093/­cercor/­1.1.62.

 2 Kaneko K, Tsuda I. Chaotic itinerancy. Chaos: An Interdisciplinary Journal of Nonlinear Science. 2003;13(3):926–936. http://­dx.doi.org/­10.1063/­1.1607783.

 3 van Leeuwen C. Chaos breeds autonomy: connectionist design between bias and baby-sitting. *Cognitive Processing*. 2008;9(2):83–92. http://­dx.doi.org/­10.1007/­s10339-007-0193-8.

 4 Tyukin I, Tyukina T, van Leeuwen C. Invariant template matching in systems with spatiotemporal coding: A matter of instability. *Neural Networks*. 2009;22(4):425–449. http://­www.sciencedirect.com/­science/­article/­pii/­S089360800900015X.

 5 Treves A, Rolls ET. Computational constraints suggest the need for two distinct input systems to the hippocampal CA3 network. *Hippocampus*. 1992;2(2):189–199. http://­dx.doi.org/­10.1002/­hipo.450020209.

 6 Bar-Gad I, Bergman H. Stepping out of the box: information processing in the neural networks of the basal ganglia. *Current Opinion in Neurobiology*. 2001;11(6):689–695. http://­www.sciencedirect.com/­science/­article/­pii/­S0959438801002707.

 7 Goldberg JH, Farries MA, Fee MS. Basal ganglia output to the thalamus: still a paradox. *Trends in Neurosciences*. 2013;36(12):695–705. http://­www.sciencedirect.com/­science/­article/­pii/­S0166223613001574.

 8 Rolls ET, Treves A. Neural networks and brain function. Oxford, New York: Oxford University Press; 1998. http://­opac.inria.fr/­record=b1094909.

 9 Rolls ET. Attractor networks. *Wiley Interdisciplinary Reviews: Cognitive Science*. 2010;1(1):119–134. http://­dx.doi.org/­10.1002/­wcs.1.

# Peer review: Referee response for version 1

**DOI:** 10.5256/f1000research.10377.r16965

**Published:** 2016-10-25

**Authors:** Stuart J. Edelstein

**Affiliations:** 1 Ecole Normale Supérieur, Paris, France

This article continues the important effort of the authors and their colleagues to advance a line of research capable of fleshing out the somewhat vague concept subsumed under the heading Neural Darwinism. While the general idea that subtle functions of the brain involve Darwinian features has persisted over the last 40 years (beginning with Changeux et al. ^1^) an up-to-date synthesis has been lacking and the authors are to be congratulated for their sustained effort to achieve this goal. Part of the difficulty encountered is related to ambiguities concerning exactly what features of neo-Darwinism so successfully applied to population genetics and molecular biology, can be imported into concepts of neuroscience. In the present effort, many terms from the classical Darwinian literature are invoked, but how they are translated from population genetics to computational neuroscience is not always clear. In this respect a glossary could be very helpful, which would include the classical definition and the neural application, as applied for example to terms such as: replication, hereditary variation, breeding, fitness, mutation, deme, generation, Lamarckian, etc. In particular, the hallmark of fitness in evolution is expansion of population size, but whether and how this criterion is applicable in neuroscience should be addressed.

 A second challenge is to define the level at which Darwinian principles are applied. More explicit attention could be given to dendritic spines (an obvious target for LTP, STDP), axonal pruning (see for example Kolodkin and Tessier-Lavigne ^2^), or neural circuitry. Cortical columns are invoked in passing, but should be evaluated in more detail. Each of these levels is stochastic in some respects, but to what extent does the variation implicit in neural Darwinism require additional mechanisms? Concerning the results presented for the various simulations performed, several valuable points were raised in the comments by Karl Friston. In addition, it would be helpful to make clearer connections with respect to the putative neuronal structures simulated. For comparison, the recent success of deep learning algorithms should be considered, in so far as they do or do not mimic Darwinian mechanisms of the brain. Moreover, since a global optimum is set for the simulations, what criteria would establish optimality in a natural Darwinian system within the brain? Finally, what is the relationship of the high number of “generations” (20,000 in Figure 4) to putative neuronal processes?

 Overall, describing neuronal activity using Darwinian terminology is a double-edged sword. On the one hand, a thorough application of Darwinian principles to brain science involves many one-to-one correspondences that must be clearly articulated. On the other hand, since the words are familiar, their usage carries immediate associations that can obscure understanding and become an ambiguous jargon. It would be hard to over-estimate the difficulty of finding the right balance, especially for scholars already immersed in the quest and possessing their own specific understanding of terms employed. Therefore, navigating through these troubled waters, requires extreme vigilance of language, and an addition effort by the authors in preparing their final version would be extremely helpful.

I have read this submission. I believe that I have an appropriate level of expertise to confirm that it is of an acceptable scientific standard, however I have significant reservations, as outlined above.

## References

[1] ChangeuxJPCourrègePDanchinA: A theory of the epigenesis of neuronal networks by selective stabilization of synapses. *Proc Natl Acad Sci U S A*.1973;70(10) :2974-8 451794910.1073/pnas.70.10.2974PMC427150

[2] KolodkinALTessier-LavigneM: Mechanisms and molecules of neuronal wiring: a primer. *Cold Spring Harb Perspect Biol*.2011;3(6) : 10.1101/cshperspect.a001727 10.1101/cshperspect.a001727 21123392PMC3098670

# Response

**Published:** 2017-06-17

**Authors:** István Zachar

**Affiliations:** ELTE, Hungary

We are extremely thankful for Stuart J. Edelstein for his comments and suggestions.

 Concerning the terms we used here to relate objects and processes from neuroscience and classical Darwinism, we have included a glossary that summarizes the important evolutionary and neurobiological concepts that might be useful to clarify across disciplines. We are certain that this is not an absolute and perfect mapping of concepts, but should be enough to support our understanding of how the brain might work under Darwinian principles.

 We further direct our Reviewer’s attention to the answers we have provided for László Acsády on the grounding of neural Darwinism within the brain (as we understand it), especially our answers to points #1, #2 about the Discussion, as those also concern the questions raised here. We simply did not want to replicate our words unnecessarily.

# Peer review: Referee response for version 1

**DOI:** 10.5256/f1000research.10377.r16922

**Published:** 2016-10-11

**Authors:** Karl Friston

**Affiliations:** 1 Wellcome Trust Centre for Neuroimaging, University College London, London, UK

This is a thought provoking simulation study of Darwinian neurodynamics. It uses populations of attractor networks to illustrate the distinction between purely selectionist and evolutionary optimisation. This demonstration rests upon the dynamical instability of the neuronal networks considered – and the explicit introduction of variation or mutations in graduating from a selectionist to an evolutionary scheme. The paper is rather dense and I do not pretend to follow all the subtleties and nuances; however, the basic ideas are compelling and are described with sufficient clarity and detail for the interested reader to understand. There are a few points of clarification that I think you could attend to. In addition, there are some minor grammatical improvements you could consider. 

**Major points**

1. I think you need to overview your simulations so that the reader knows where you are going. I would recommend something like:
  “We will present a series of simulations graduating from purely selectionist schemes to evolutionary schemes in the face of a changing environment. In the first set of simulations we preclude variation in transmission over generations to examine the sufficiency of dynamical instabilities in supporting a selective process. This involves taking the outputs of one neural network and using them (after selection) to train another network. In the second set of simulations, we consider evolution proper and the transmission of patterns from generation to generation. Here, the patterns that are transmitted are subject to mild mutations (and selection) to illustrate the efficiency with which optimal (high adaptive fitness) patterns emerge.”
2. I think you need to explain simply what is being optimised in your simulations. I would suggest something like: 
  “In what follows, we will treat a pattern of activations over binary neurons as the unit of selection. In the general case, the adaptive fitness of this pattern may be some complicated function that is contextualised by the current inputs and may or may not be a function of the history of inputs and outputs. To keep things simple, we will just consider the fitness of a pattern in terms of its hamming distance to some target pattern. This means, we are effectively using selectionist and evolutionary schemes to optimise the connections (and ensuing dynamics) to recover a target pattern.”
3. When talking about the utility of dynamical instability in providing a basis for selection, you might want to refer to the work of Ivan Tyukin and colleagues ^1^, ^2^. These authors have studied chaotic systems in the context optimisation – and their neuronal counterparts.
4. At the end of your discussion, I think you can usefully pursue the Bayesian brain hypothesis. I would suggest something like:
  “In fact, there may be a deep connection between the selectionist dynamics illustrated in this paper and the Bayesian brain. This follows from the fact that the Bayesian brain can use Bayesian model selection to identify its most plausible hypotheses about the world. In this sense, the selective mechanisms we have demonstrated become Bayesian model selection, if we use marginal likelihood or variational free energy as adaptive fitness. See for example Sella and Hirsh, 2005 ^3^ and Friston, 2013 ^4^. Crucially, the evolutionary role of mutations and variations provides the extra ingredient required for structure learning; namely the elaboration of a model or hypothesis space."

**Minor points**

 Page 3:

- Replace "Bayesian update" with "Bayesian updates". 
- You might want to add a footnote about reentry and neural Darwinism when you say that “this fast scale dynamics is missing from Edelmanian neural Darwinism.” I suspect that Edelman considered variation an important aspect of neural Darwinism and that this was mediated by reentrant dynamics that shows the dynamical and structural instabilities that you refer to.  

Page 5 and throughout:

- Replace "was trained only to" with "was presented only to". 

Page 5:

- Replace "this allows finding the global" with "this allows the system to find". 
- Replace "system above the", with "system above and beyond the". 
- I would say “… can quickly converge to the optimum – providing the output of each network is delivered to the appropriate network that successively converges on the global optimum.”.

Page 6:

- Replace "at start" with "at the start".
- I would remove the simulations based upon the simpler model (i.e. the thin lines in Figure 3). These simulations and their description do not add much to the text – or any insight. It would be less distracting if these simulations and their discussion were removed.

Page 8 and throughout:

- Replace "provoking them" with "perturbing them". 
- Replace "performed of" with "performed in". 

Page 8:

- It would be useful to mention the (Stuart Kauffman) notion of second order selection and selection for selectability (or evolvibility). In other words, you should discuss the optimisation of the mutation rates in relation to the volatility of the environment. 

Page 9:

- Replace "solves the problem" with "solving the problem". 

Page 10:

- Replace "networks in the given" with "networks under the given". Later replace "attractor network" with "attractors networks". Later replace "works with diluted" with "work with diluted". Finally, replace "explained in turn" with "explained next". 

 

 I hope that these comments help should any revision be required.

I have read this submission. I believe that I have an appropriate level of expertise to confirm that it is of an acceptable scientific standard.

## References

[1] TyukinITyukinaTvan LeeuwenC: Invariant template matching in systems with spatiotemporal coding: A matter of instability. *Neural Netw*.2009;22(4) : 10.1016/j.neunet.2009.01.014 425-49 10.1016/j.neunet.2009.01.014 19264447

[2] van LeeuwenC: Chaos breeds autonomy: connectionist design between bias and baby-sitting. *Cogn Process*.2008;9(2) : 10.1007/s10339-007-0193-8 83-92 10.1007/s10339-007-0193-8 17924155

[3] SellaGHirshAE: The application of statistical physics to evolutionary biology. *Proc Natl Acad Sci U S A*.2005;102(27) : 10.1073/pnas.0501865102 9541-6 10.1073/pnas.0501865102 15980155PMC1172247

[4] FristonK: Life as we know it. *J R Soc Interface*.2013;10(86) : 10.1098/rsif.2013.0475 20130475 10.1098/rsif.2013.0475 23825119PMC3730701

# Response

**Published:** 2017-06-17

**Authors:** István Zachar

**Affiliations:** ELTE, Hungary

We are thankful for Karl Friston for his invaluable comments and suggestions. Please also see our answers to the other Reviewers for some relevant answers.

About reentry and neural Darwinism: We previously discussed the limitations of Edelman’s so-called “neural Darwinism” [1] in the light of standard evolutionary dynamics [2]. Here we consider two important aspects: the roles of variation and selection and reentry [1,3] and how these relate to our model. In general, there are no units of evolution in the Edelman model, only units of selection. Edelman considered three subsequent phases of brain development and function (i) developmental variation and selection (supported by production and elimination of neurons and their connections), (ii) experiential selection (resting on changes in the strength of a population of synapses) and (iii) reentry (where some connections between two or more maps become stronger due to changes in synaptic weights). Note that beyond stage (i) there is no real novelty emerging. The situation is more like that in combinatorial chemistry where functional molecules are selected from a large pool of generated variants. Crucially, there is no replication with variation in this picture. Reentry has two different functions in Edelman’s picture: the linking of different maps and a critical contribution to consciousness. We further direct Friston’s attention to the answer we have provided for László Acsády about the realization of reentry in the cortex-basal ganglia-thalamus-cortex loop.

Points 1, 2. We have opted to borrow your expressions of Points 1 and 2 that perfectly summarize our original intention, and included them within the Methods section to introduce our selectionist and evolutionary experiments.

Point 3. Chaotic itinerancy is very different from our model. Our search uses the overlaps between attractors in different networks and is guided by evaluation of interim findings. Darwinian neurodynamics harnesses neuronal parallelism for search more profoundly than chaotic itinerancy.

Point 4. We have added an explanation highlighting the connection between selectionist dynamics and the Bayesian brain. We additionally cite Iwasa, who pioneered the concept as well as Barton & de Vladar who refined it (and it would be odd not to cite some of the work of de Vladar, a co-author of this paper, who merged and developed further the ideas of Iwasa and Sella & Hirsch).

Considering Figure 3. and the suggestion to remove the simpler model, we decided rather not to modify this section. Actually, thin lines represent the behaviour of the complex attractor network-model introduced in the paper and thick lines represent the simpler model that superficially approximate attractor dynamics without actually using networks. The whole point of this image is the comparison between our proof-of-principle attractor network model and a baseline, abstracted model.

Certainly to Stuart Kauffman’s work is an important aspect that is present in our studies. We added some explanations in this direction. However, note that the notion of evolvability is not Kauffman’s (although he certainly did a good work on it and on its popularisation), but is attributed, under that name, to Dawkins and back to Riedl (1975) (but not using that term).

Most of the minor points were corrected except in a few cases where they went explicitly against our intended meaning. For example we rather stick with the word "provocation", as "perturbation" has a different meaning to what we intended here.

References:

1 Edelman GM. Neural Darwinism. The theory of neuronal group selection. New York: Basic Books; 1987.

2 Fernando C, Szathmáry E, Husbands P. Selectionist and evolutionary approaches to brain function: a critical appraisal. *Frontiers in Computational Neuroscience*. 2012;6(24). http://­dx.doi.org/­10.3389/fncom.2012.00024.

3 Edelman GM. Neural Darwinism: Selection and reentrant signaling in higher brain function. *Neuron*. 1993;10(2):115–125. http://­www.sciencedirect.com/­science/­article/­pii/­089662739390304A.
